# Supplementary material for: Application of the Transition State Theory in the Study of the Osmotic Permeabilities of AQP7, AQP10 and GlpF
Source: Membranes (Basel). 2025 Sep 2;15(9):265. doi: 10.3390/membranes15090265 (PMC12471668; doi:10.3390/membranes15090265)
Supplement: Supplementary file 1 [file membranes-15-00265-s001.zip › membranes-3740347-supplementary.pdf]

Supplemental Information

Application of the Transition State Theory in the Study of the Osmotic Permeabilities of AQP7, AQP10 and GlpF

Ruth Chan[1], Liao Y. Chen[1*]

[1]Department of Physics, The University of Texas at San Antonio, San Antonio, Texas 78249 USA

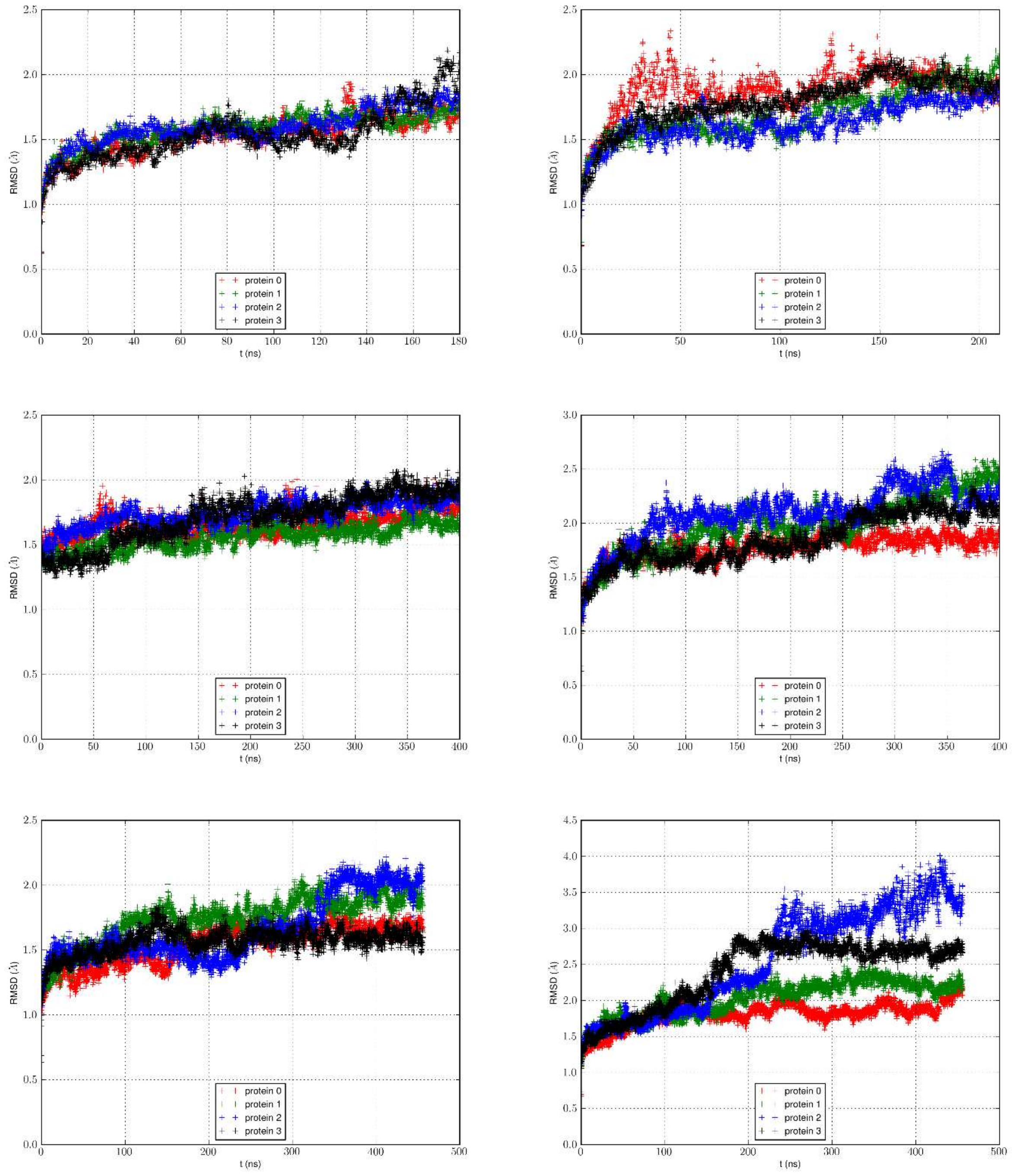

Figure S1. RMSD of the individual monomers in the aquaglyceroporin protein tetramers for the duration of the MD simulations. From left to right, the results shown are 5°C followed by 25°C. The top row is for AQP7, followed by GlpF, then AQP10.

Table S1 Average RMSF values of all the residues in the NPA motifs and selectivity filters respectively, for AQP7, AQP10 and GlpF.

	**RMSF**			
	**NPA motifs (Å)**		**Selectivity Filter (Å)**	
	**5°C**	**25°C**	**5°C**	**25°C**
**AQP7**	0.423 ± 0.054	0.492 ± 0.063	0.465 ± 0.050	0.718 ± 0.086
**AQP10**	0.436 ± 0.061	0.486 ± 0.066	0.466 ± 0.053	0.508 ± 0.062
**GlpF**	0.413 ± 0.053	0.463 ± 0.064	0.488 ± 0.058	0.594 ± 0.070

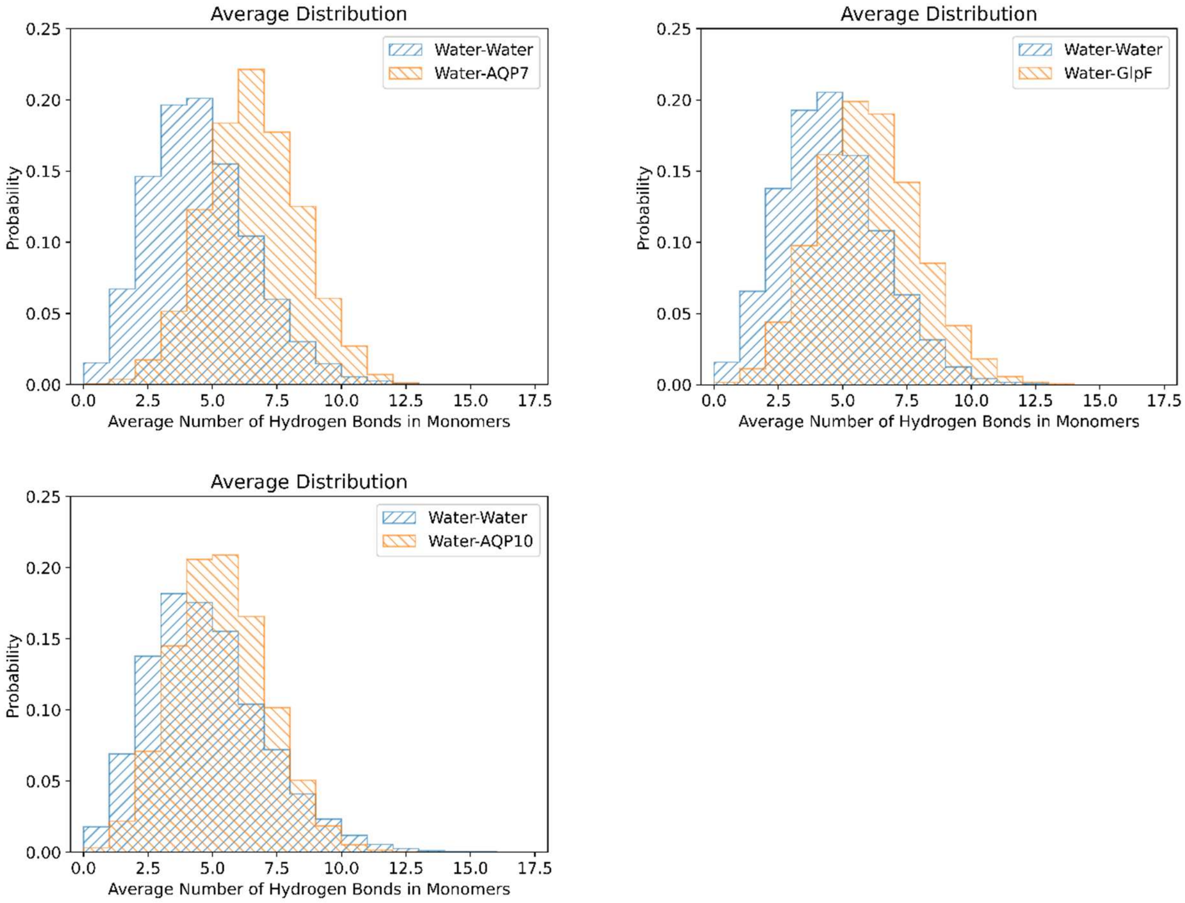

Figure S2. Average probability distribution for all monomers of AQP7, GlpF and AQP10 respectively of hydrogen bonds between the water molecules (in blue) and between water molecules and channel residues (in orange) at 5°C.

AQP7 at 25°C

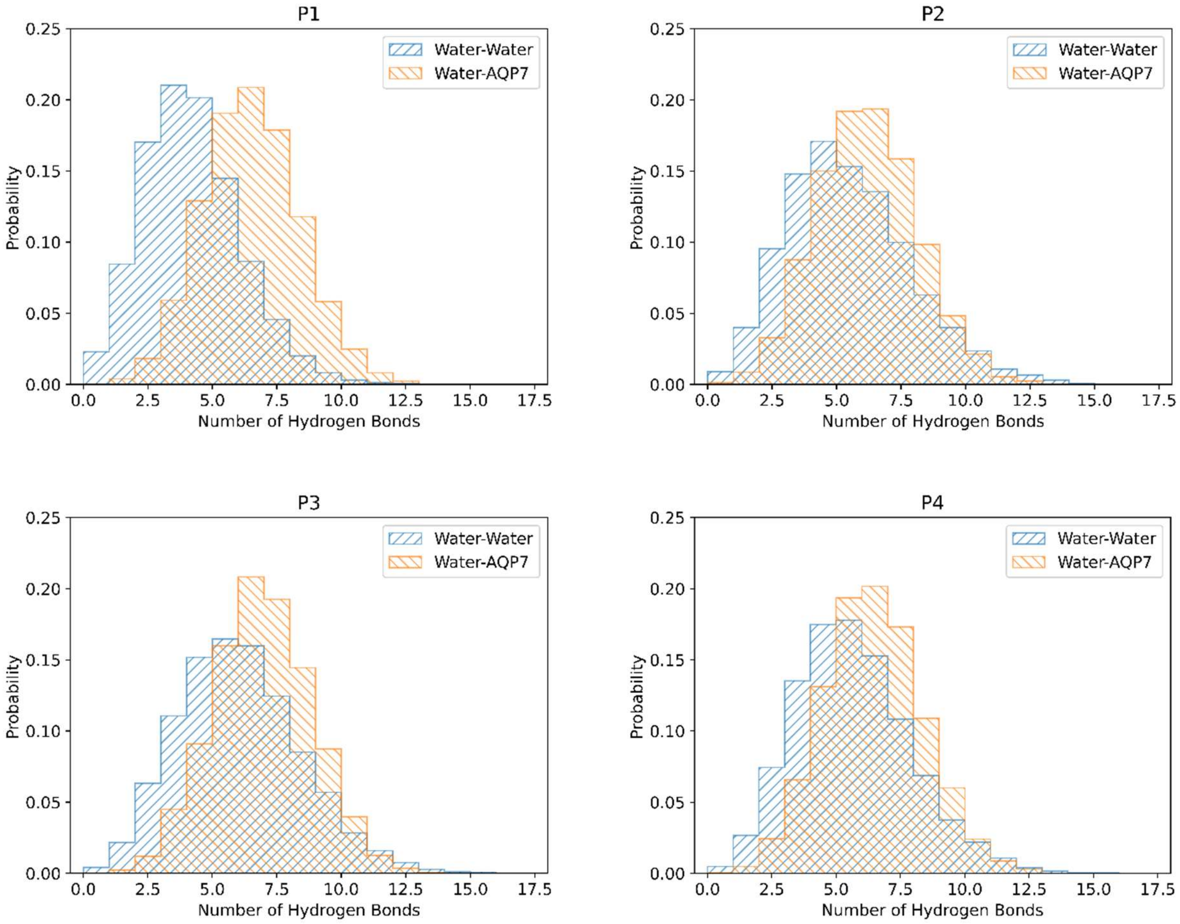

Figure S3. Probability distribution of the hydrogen bonds of individual monomers (P1 – P4) between water molecules (in blue) and between water molecules and channel residues (in orange), for AQP7 at 25° C.

GlpF at 25°C

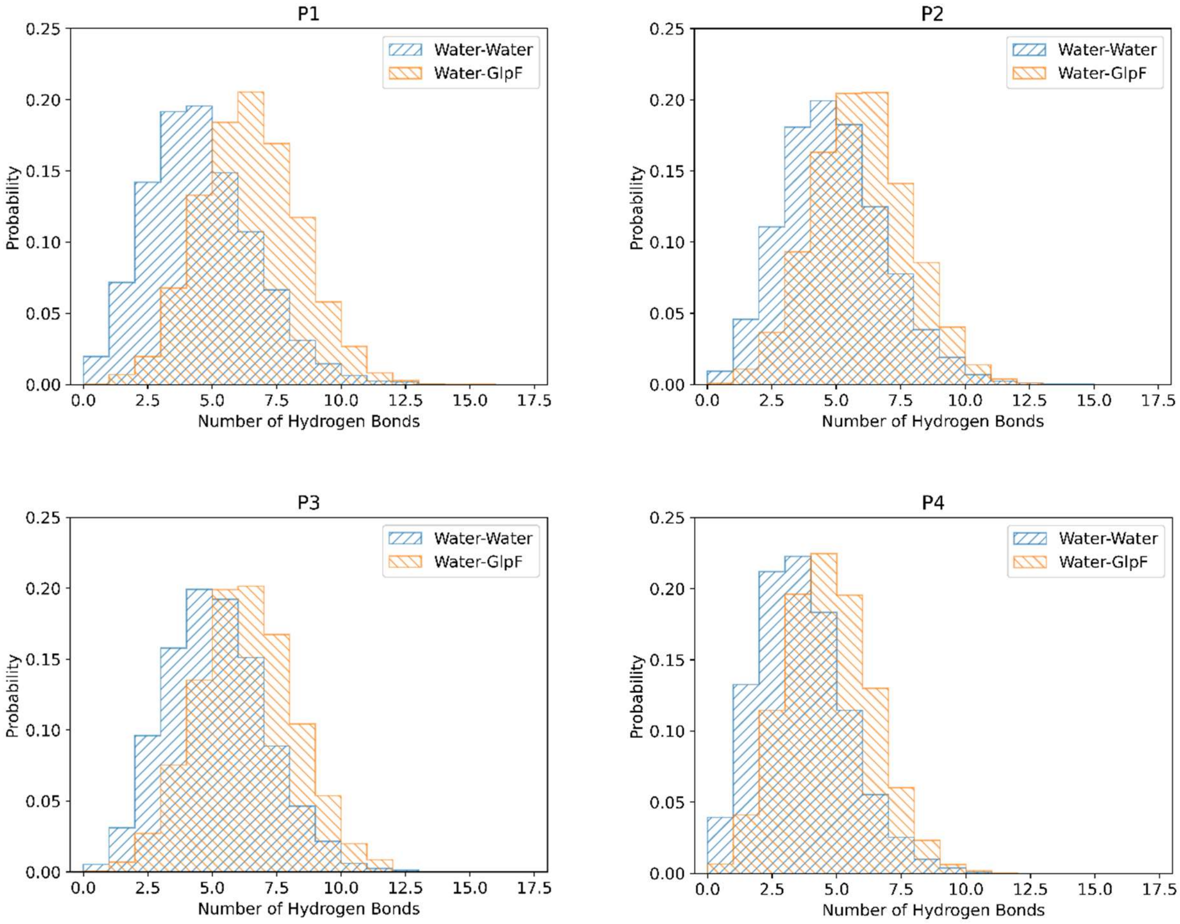

Figure S4. Probability distribution of the hydrogen bonds of individual monomers (P1 – P4) between water molecules (in blue) and between water molecules and channel residues (in orange), for GlpF at 25°C.

AQP10 at 25°C

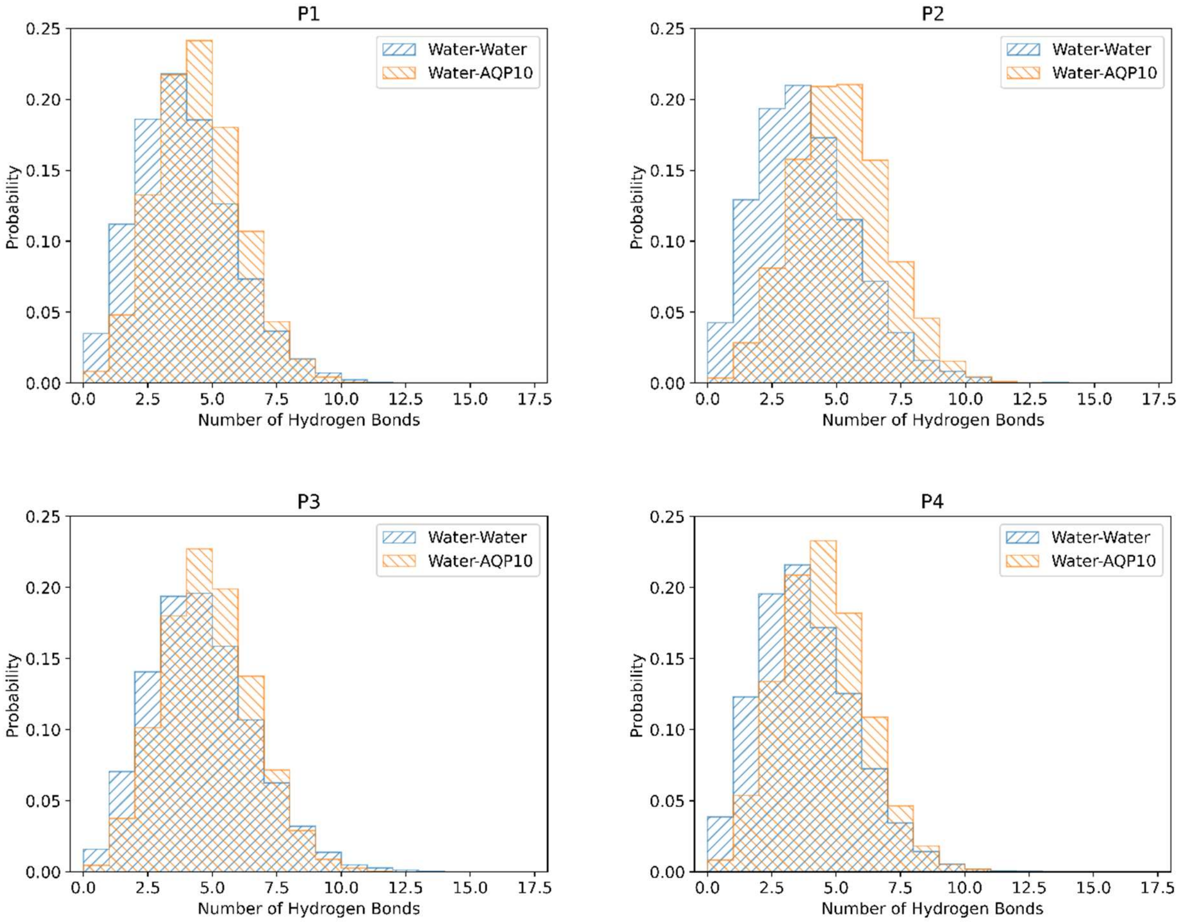

Figure S5. Probability distribution of the hydrogen bonds of individual monomers (P1 – P4) between water molecules (in blue) and between water molecules and channel residues (in orange), for AQP10 at 25°C.

AQP7 at 5°C

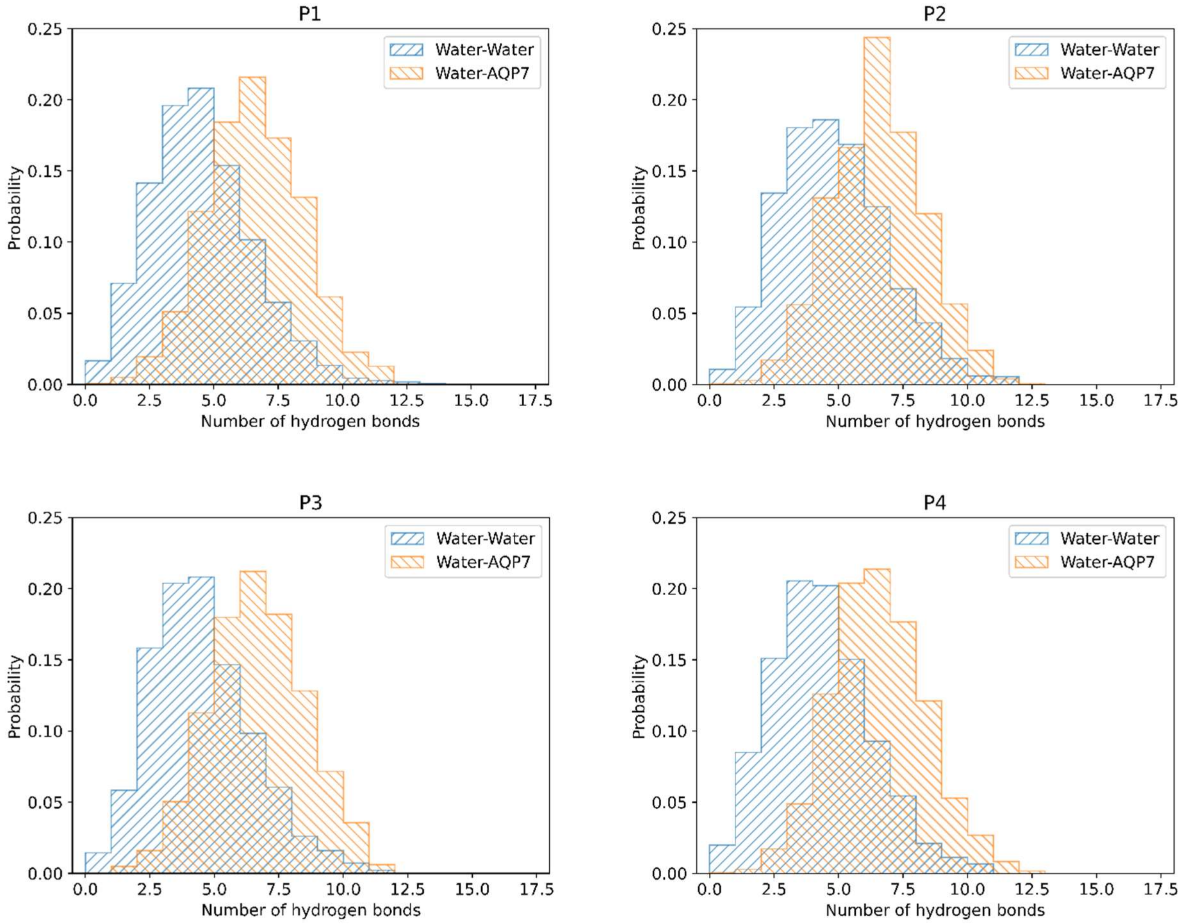

Figure S6. Probability distribution of the hydrogen bonds of individual monomers (P1 – P4) between water molecules (in blue) and between water molecules and channel residues (in orange), for AQP7 at 5°C.

GlpF at 5°C

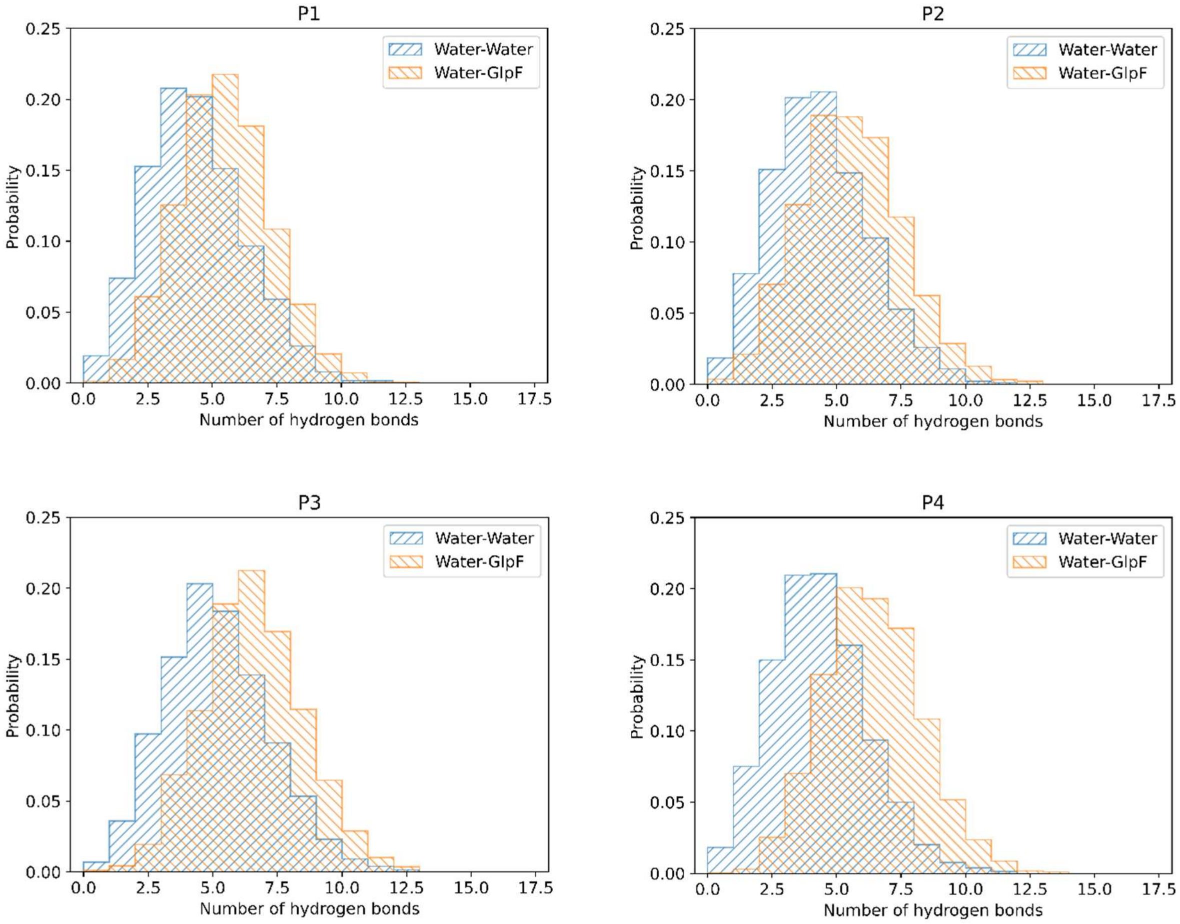

Figure S7. Probability distribution of the hydrogen bonds of individual monomers (P1 – P4) between water molecules (in blue) and between water molecules and channel residues (in orange), for GlpF at 5°C.

AQP10 at 5°C

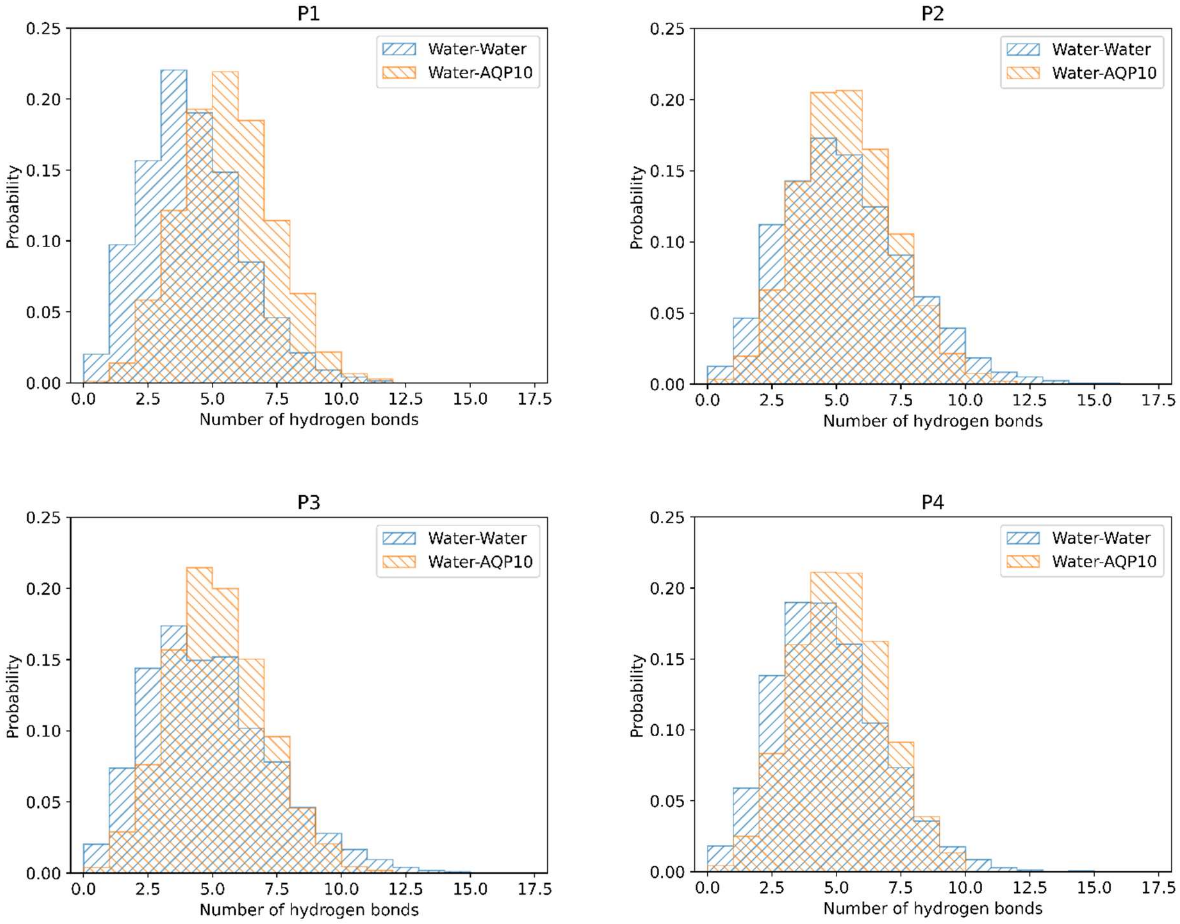

Figure S8. Probability distribution of the hydrogen bonds of individual monomers (P1 – P4) between water molecules (in blue) and between water molecules and channel residues (in orange), for AQP10 at 5°C.

5°C **25°C**

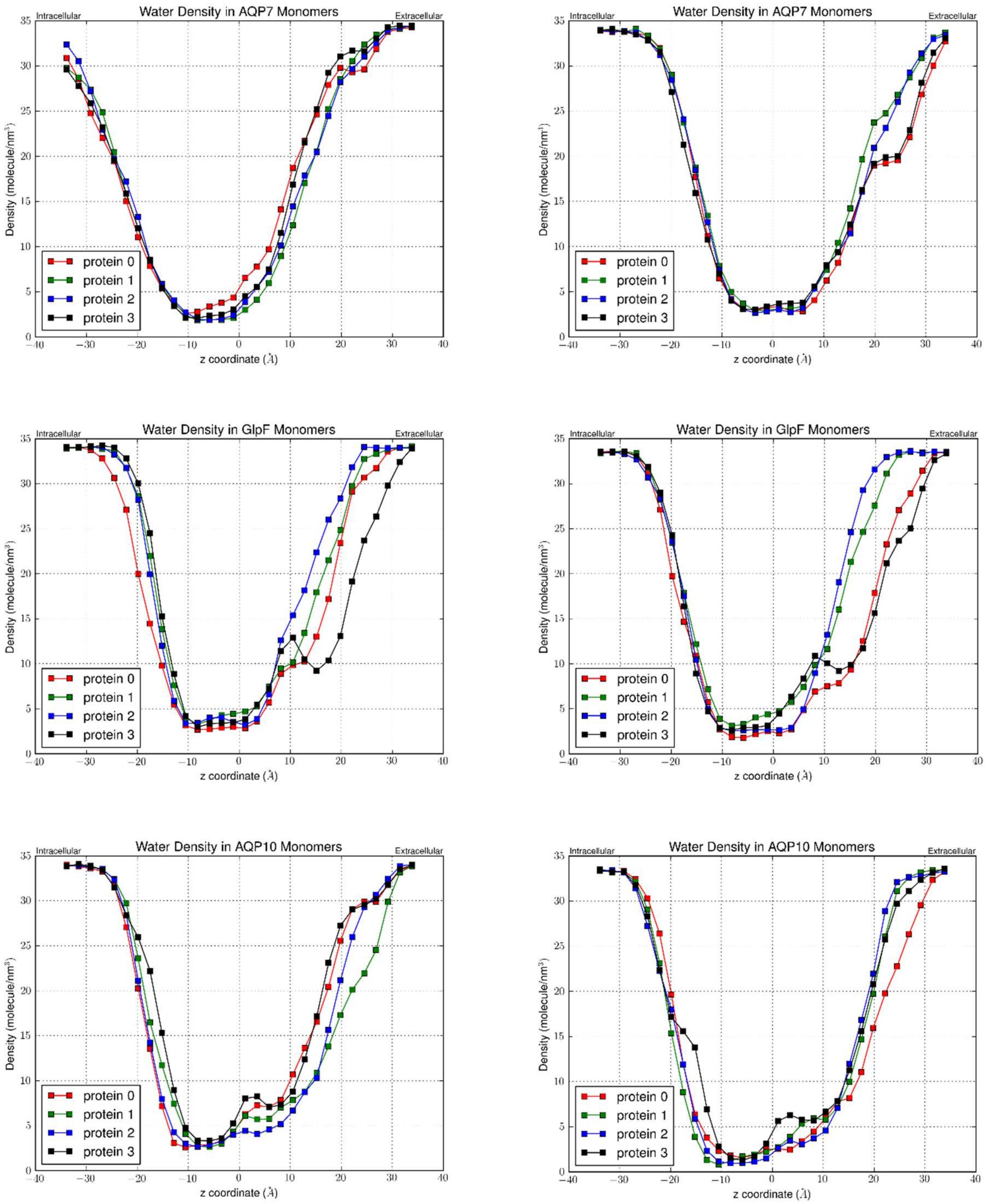

Figure S9. Linear density of water molecules throughout the channel length parallel to the z-axis for the individual monomers of AQP7, GlpF and AQP10 at 5°C and 25°C.

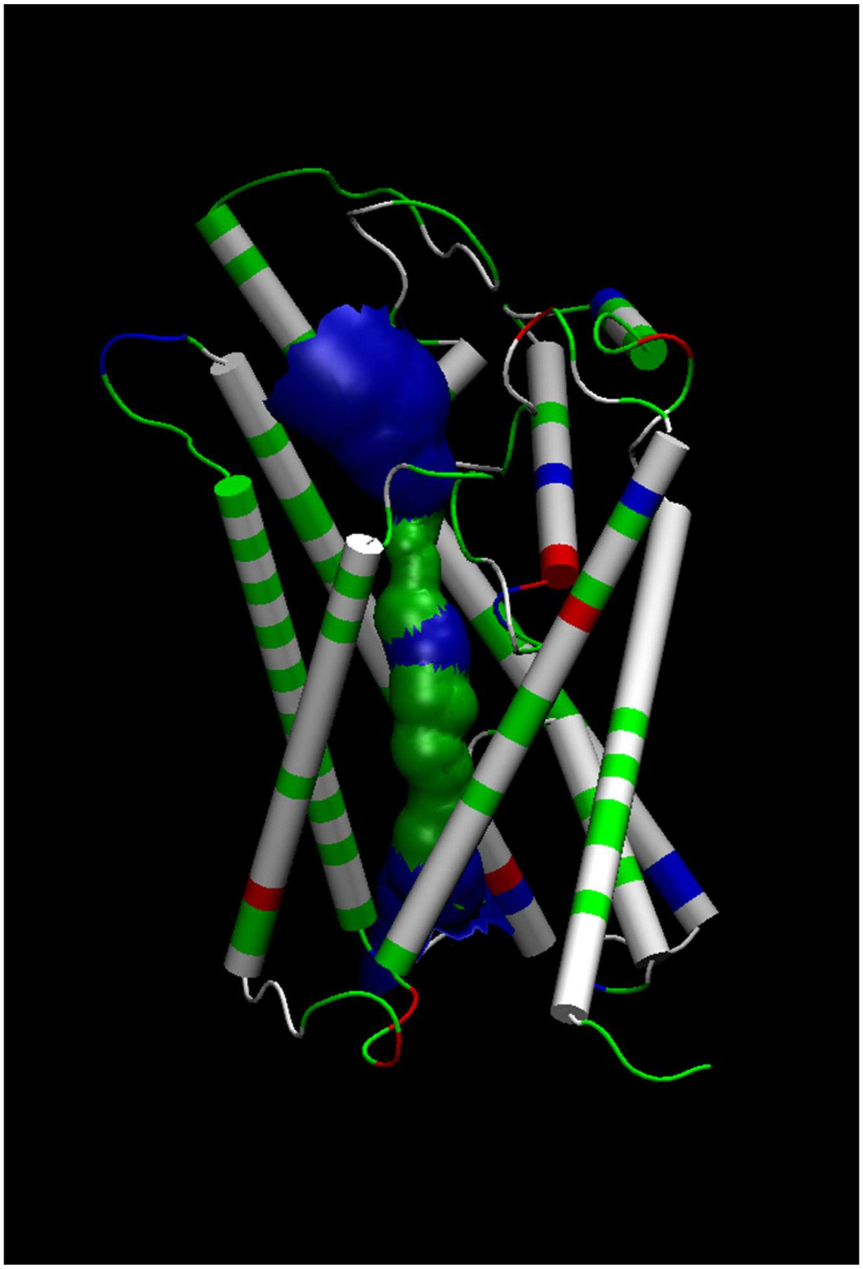

Figure S10. AQP7 channel illustrated in HOLE representation. The protein monomer is also illustrated in Cartoons colored by residue types (hydrophilic, green; hydrophobic, white; negatively charged, red; positively charged, blue).

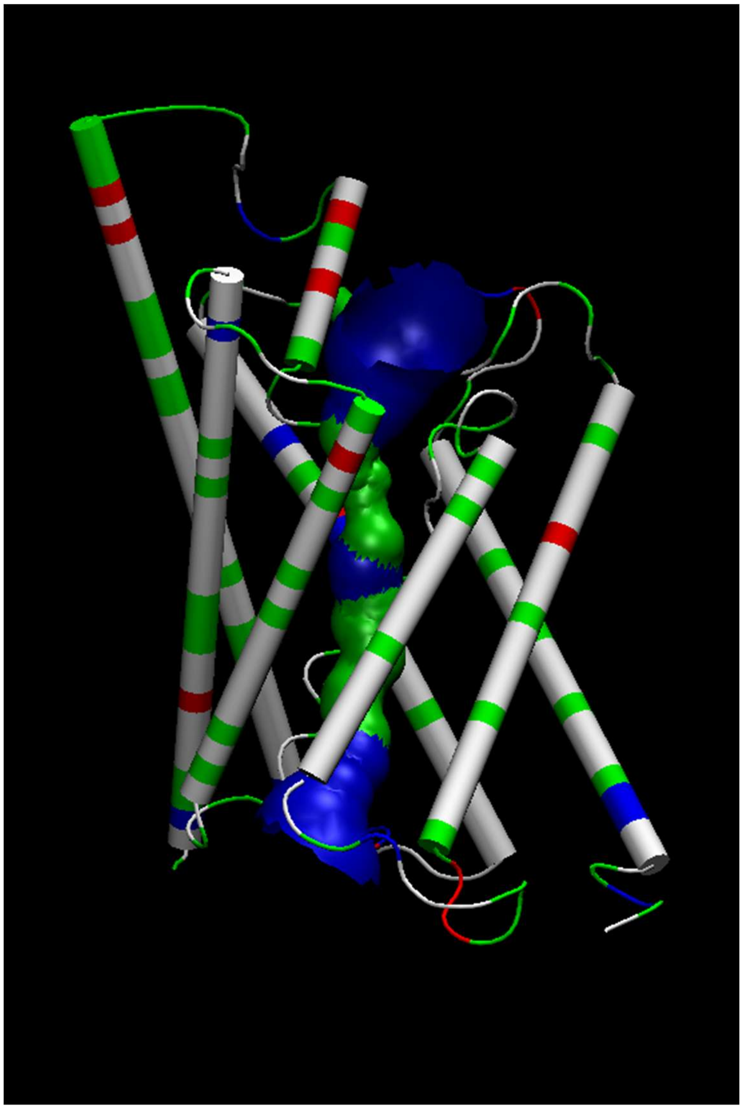

Figure S11. GlpF channel illustrated in HOLE representation. The protein monomer is also illustrated in Cartoons colored by residue types (hydrophilic, green; hydrophobic, white; negatively charged, red; positively charged, blue).

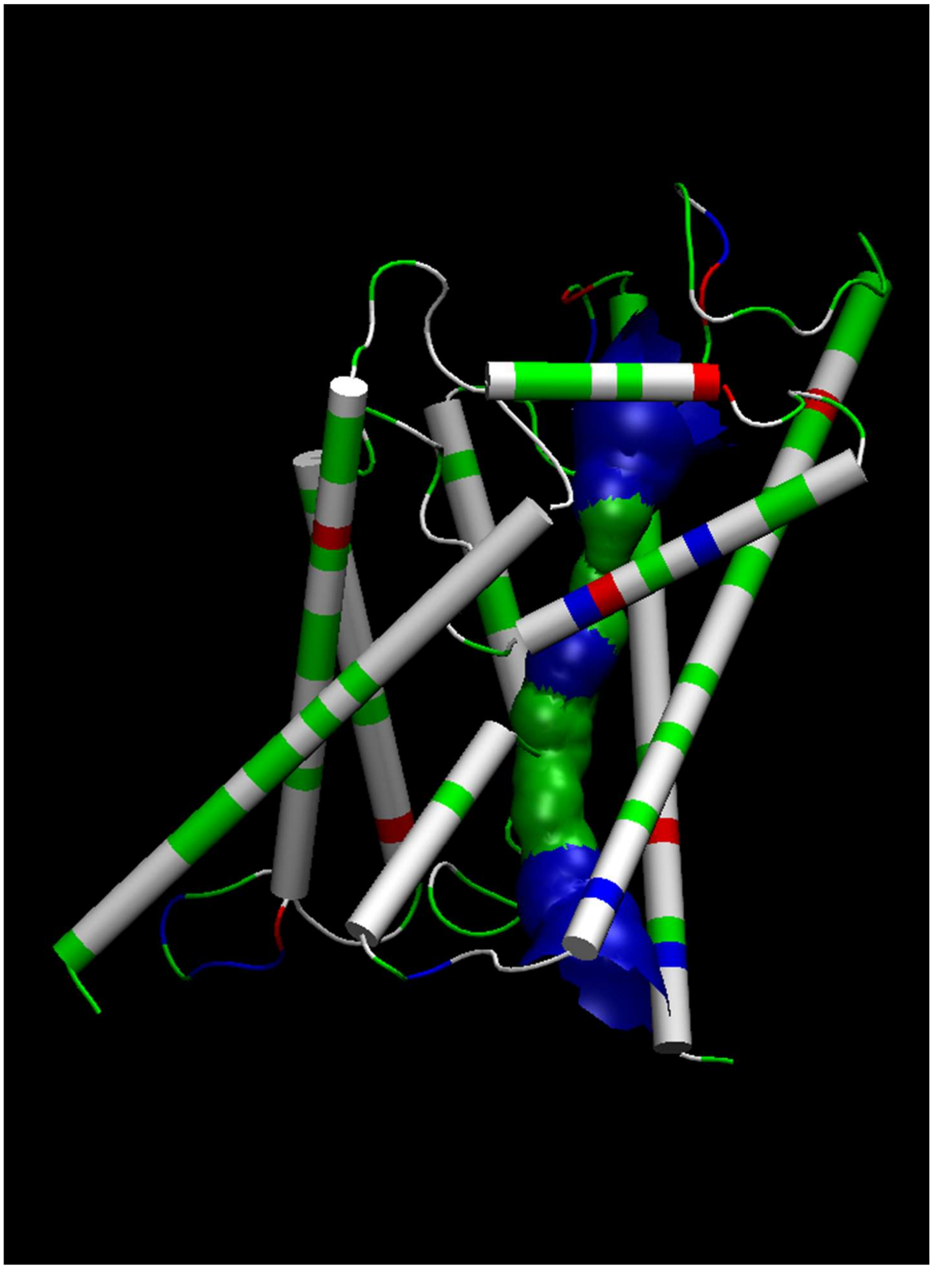

Figure S12. AQP10 channel illustrated in HOLE representation. The protein monomer is also illustrated in Cartoons colored by residue types (hydrophilic, green; hydrophobic, white; negatively charged, red; positively charged, blue).
